# Biomechanical evaluation of different sizes of 3D printed cage in lumbar interbody fusion-a finite element analysis

**DOI:** 10.1186/s12891-023-06201-7

**Published:** 2023-02-01

**Authors:** Jincheng Wu, Qing Feng, Dongmei Yang, Hanpeng Xu, Wangqiang Wen, Haoxiang Xu, Jun Miao

**Affiliations:** 1 Department of Spine Surgery, Tianjin Hospital, Tianjin University, Jiefangnanlu 406, Hexi District, Tianjin, China; 2 Southern Medical University, Guangzhou City, Guangdong China; 3 The First Affiliated Hospital of Hainan Medical University, Haikou City, Hainan China; 4 The Second People’s Hospital of Hefei, Hefei, Anhui China

**Keywords:** Lumbar interbody fusion, 3D printed cage, Cage subsidence, Spine, Non-union, Finite element analysis

## Abstract

**Objective:**

To study the biomechanical characteristics of various tissue structures of different sizes of 3D printed Cage in lumbar interbody fusion.

**Methods:**

A finite element model of normal spine was reconstructed and verified. Pedicle screws and Cage of different sizes were implanted in the L4/5 segment to simulate lumbar interbody fusion. The range of motion of the fixed and cephalic adjacent segment, the stress of the screw-rod system, the stress at the interface between cage and L5 endplate, and intervertebral disc pressure of the adjacent segment were calculated and analyzed.

**Results:**

The range of motion and intervertebral disc pressure of the adjacent segment of each postoperative model were larger than those of the intact model, but there was not much difference between them. The stress of cage-endplate interface was also larger than that of the intact model. However, the difference is that the stress of the endplate and the screw-rod system has a tendency to decrease with the increase of the axial area of cage.

**Conclusions:**

Cage with larger axial area in lumbar interbody fusion can reduce the stress of internal fixation system and endplate, but will not increase the range of motion and intervertebral disc pressure of adjacent segment. It has a certain effect in preventing the cage subsidence, internal fixation system failure and screw rod fracture.

## Introduction

According to the Global Burden of Diseases, Injuries, and Risk Factors Study 2017 (GBD 2017) assessment of 359 diseases between 1990–2017, low back pain has been found to be one of the leading causes of years lived with disability (YLDs) in recent decades, which not only increases the social burden, but also seriously affects people’s quality of life [1]. The causes of low back pain are complex, and the concept for explaining backache, “biopsychosocial pain syndrome,” has been put forward, in which biological factors include spinal morphological abnormalities and spinal mechanical dysfunctions, such as intervertebral disc degeneration, nerve compression, bacterial infection or inflammatory diseases, facet joint disorders, vertebral fractures, malignant tumors and neuromuscular diseases [2–5].

As for the treatment of low back pain, we generally choose conservative treatment. However, we consider surgical treatment for patients with failed conservative treatment, moderate / severe persistent pain, cauda equina syndrome and severe neurological symptoms [6]. Interbody fusion plays an important role in it. Different surgical approaches, implants and grafts can achieve biomechanically lasting interbody union, so as to stabilize the degenerative segments, restore the physiological curvature of the spine and decompression of the spinal canal to achieve the purpose of treatment [7, 8]. However, interbody fusion will not only accelerate the degeneration of adjacent segments and lead to the occurrence of adjacent segment disease (ASD), but also may occur complications such as cage subsidence, failed solid bony fusion, displacement and so on [4, 8]. Cage subsidence rates have been reported to range from 15.9% to 70%, with rates of non-union or pseudarthrosis ranging from 5 to 35%, and even higher rates of non-union in patients spanning 3 or more spinal segments [9, 10].

According to the latest report, the material of cage does not affect bone fusion. and there is no significant difference in the fusion rate between cages made of PEEK, titanium, tantalum and other materials [11]. The ideal biological agents with osteoinductive, osteoconductive and osteogenic properties can improve the success rate of bone fusion [11, 12]. Cage subsidence has attracted more and more attention because it may lead to the reduction of the height of fusion segment, changes in spinal curvature and possible nerve compression, which may affect the prognosis of patients, such as recurrent low back pain and related nervous system diseases [10, 13]. Some studies have analyzed and reported the risk factors of cage subsidence [10, 13, 14], but there are few reports on the effect of different contact area between cage and endplate on cage subsidence, including a recent experimental study on the correlation between cage subsidence and different sizes of cage of TLIF and LLIF [15]. However, as far as we know, there is no biomechanical analysis of the different contact area between cage and endplate during lumbar interbody fusion.

Finite element (FE) analysis does not require the necessary in vitro test equipment and various specimens and can change the geometry of the model according to the design needs. It can reflect the interaction between various structures more accurately. At the same time, it allows direct comparison of experimental conditions to avoid the influence of individual differences between experimental materials on experimental results, and improves the accuracy of the analysis results [16]. FE analysis has been applied to the biomechanical field of skeletal parts since 1972 [17]. With the improvement of computing power over the years, it is widely used in the medical field, because it can predict function, behavior and possible complications through model [18]. It provides us with information that cannot be provided by laboratory experiments, and avoids the limitations of time-consuming, difficulties in parameter modification, and allowance of only generalized results [19, 20].

With the development of 3D printing technology, the personalized design of 3D printing cage can ensure a close fit with the vertebral body, and its microporous structure is conducive to bone fusion [21]. Therefore, this study uses the FE analysis to evaluate the mechanical characteristics of different types of 3D printed cage in lumbar interbody fusion, in order to provide reference for the selection of cage in clinical practice.

## Materials and methods

### Intact FE model

Data of the L1-S lumbar spine FE model were collected from a healthy adult male volunteer (24 years old, weight 67 kg, height 173 cm). The volunteer had no previous history of trauma or fracture. Any spinal diseases were excluded by clinical imaging examination to establish a normal intact FE model. The volunteer was recruited by the Spinal Surgery Department of Tianjin Hospital and signed informed consent forms in accordance with the relevant regulations, which were submitted it to the Ethics Committee of Tianjin Hospital for approval. All clinical investigations were conducted in strict accordance with the principles of the Declaration of Helsinki.

A 64-slice spiral computed tomography scanner (Siemens, Erlangen, Germany) was used to obtain tomographic data in DICOM format with a slice spacing of 0.625 mm, which included imaging data of one sacrum and five vertebral bodies and intervertebral discs. The method of model reconstruction was consistent with the previous study [22]. The image data were imported into Mimics20.0 (Materialise Inc., Leuven, Belgium), and the 3D geometric surface model of the lumbar spine was reconstructed and saved in STL format [23]. The model was imported into 3-Matic 12.0 software (Materialise Inc.) to perform wrapping, smoothing and Boolean operation. The redundant triangular surfaces were removed to generate more detailed 3D images, and the structures of facet joints, intervertebral discs and nucleus pulposus were initially constructed [24]. The 3D surface model of lumbar vertebrae was imported into Hypermesh 2017 (Altair Engineering, Troy, Michigan, USA) after smoothing and accurate surface processing with Geomagic Studio 12.0 (Geomagic Inc., USA), and seven ligaments were constructed (ALL: anterior longitudinal ligament; PLL: posterior longitudinal ligament; LF: ligamentum flavum; CL: capsular ligament; ISL: interspinous ligament; SSL: supraspinous ligament; ITL: Intertransverse ligament). Finally, the model was imported into Abaqus 2019 (Simulia, Johnston, RI, USA) for assembly, material property definition and FE analysis [25, 26].

**Fig. 1:**
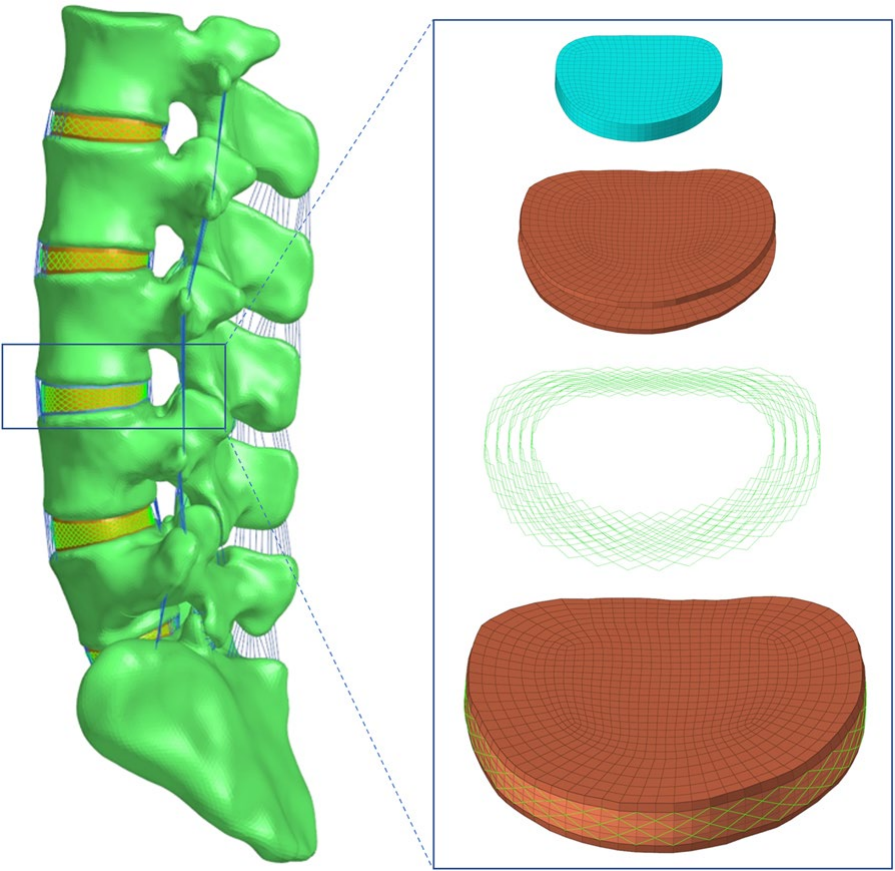
Finite element models of the intact lumbar spine and intervertebral disc structures

In this study, we reconstructed a three-dimensional FE model (Fig. 1) of the normal L1-S lumbar vertebrae. The intervertebral disc uses hexahedral mesh, which is composed of annulus ground substance, nucleus pulposus, annulus fibers and cartilaginous endplate. The thickness of the upper and lower endplate is 0.5 mm [27], and the nucleus pulposus accounts for 30%-40% of the total disc [28–30]. Ligaments and annulus fibers were simulated by using tension-only truss elements, in which fibers were constructed from inside to outside and embedded into the annulus ground substance at an angle of ± 30°. The elastic strength increased proportionally from the innermost (360 MPa) to the outermost fibers (550 MPa) [28, 31, 32]. The vertebral body was composed of cortical, cancellous and posterior bone structures, which were divided by tetrahedral mesh. The thickness of cortical bone and articular cartilage was 1 mm and 0.2 mm respectively [23, 27]. The intact L1-S model contained 1,489,577 elements and 370,061 nodes, and the material properties were defined according to the previously reported literature (Table 1) [27, 28, 33, 34].

**Table 1:** Material properties used by finite element model

Component	Young’s Modulus (MPa)	Poisson Ratio	Cross-Sectional Area(mm^2^)
Vertebra			
Cortical bone	12,000	0.3	
Cancellous bone	100	0.2	
Posterior element	3500	0.25	
Sacrum	5000	0.2	
Facet	11	0.2	
Disc			
Endplate	24	0.4	
Nucleus pulpous	1	0.49	
Annulus ground substance	2	0.45	
Annulus fibers	360–550		0.15
Ligaments			
ALL	7.8		63.7
PLL	10		20
LF	15		40
CL	7.5		30
ISL	10		40
SSL	8		30
ITL	10		1.8
Implants			
Cage (titanium alloy)	110,000	0.3	
Porous part of cage	675	0.3	
Bone graft	100	0.2	
Screws and rods (titanium alloy)	110,000	0.3	

### Model simulation

Lumbar degenerative diseases often occur in the L4/5 segment, so it was selected for posterior lumbar interbody fusion (PLIF) in this study. According to the commonly selected resection structure and surgical decompression range of PLIF surgery, the posterior part of spinous process and lamina of L4 vertebral body, the medial 1/2 of bilateral L4 inferior articular process and L5 superior articular process, and the corresponding SSL, ISL, LF and CL were removed. At the same time, the L4/5 segmental intervertebral disc was also removed.

In this study, the internal fixation instruments required for the simulated surgery were constructed Pro/Engineer 5.0 software. In order to obtain a convenient internal fixation model, combined with previous studies and PLIF operation, a simplified lumbar pedicle screw (diameter 6.5 mm, length 45 mm) and connecting rod (diameter 5.5 mm) were designed. Because the sliding of the screw in the vertebral body was not considered, we removed the screw thread in order to simplify the analysis without affecting the results of the study we were concerned about [35, 36]. Two pedicle screws were placed in L4 and L5 vertebrae, respectively. The insertion point was the intersection of the outer edge of the articular process and the midline of the transverse process. Tie contact was used at the screw and link junctions to form a rigid connection between them [37, 38]. Similarly, the same constraint was used between the pedicle screws and the vertebral body, limiting the relative movement between the screw and the vertebral body [38, 39].

**Fig. 2:**
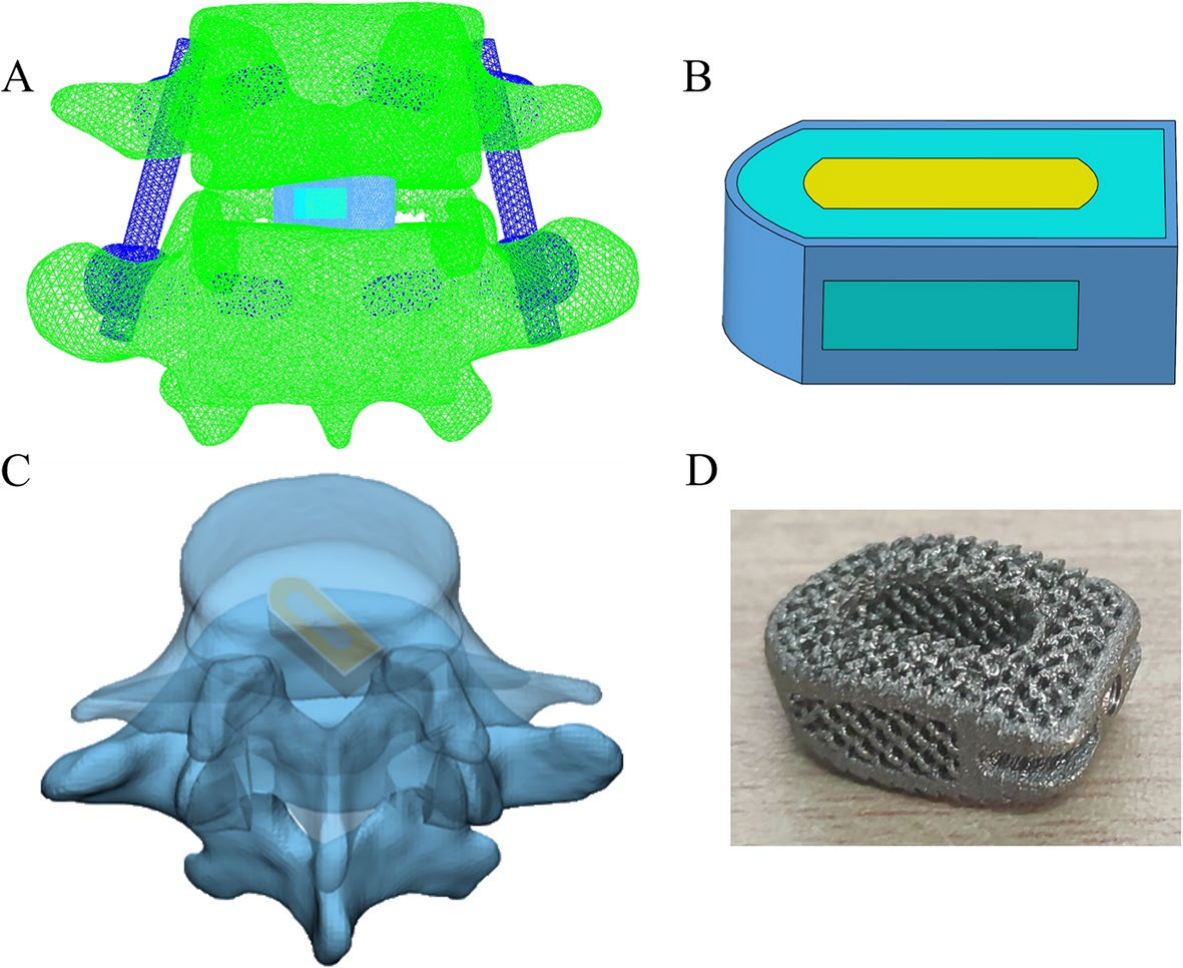
**A**, **C** Schematic diagram of the operative segment. **B** Finite element model of cage. **D** the product of the 3D printed cage

Proe software was used to draw three different sizes of Cage (cage A: 9 × 10 × 23 mm, cage B: 9 × 10 × 26 mm, cage C: 15 × 10 × 23 mm), all of which were made of titanium alloy. Models A, B, and C were surgical models with cages of different sizes implanted into the L4/5 intervertebral space, respectively (Fig. 2). To simulate graft fusion under internal fixation, we filled all cage graft holes with cancellous bone. We applied Boolean operation to remove the part of the cage in contact with the vertebral body and the geometric contact between the Cage and the vertebral body was satisfied. And the interface assigns a friction coefficient of 0.2 to simplify the effect of the teeth of cage [40]. In order to simplify the analysis and reduce the calculation cost, referring to the previous experimental method, we adopted small Young’s modulus to simulate the porous part of cage [41].

### FE model validation

The rationality of the intact model was verified by referring to the previous research methods of Renner et al. [42]. As with previous research methods, we fixed the bottom of the sacrum, limiting its displacement and rotation in all directions. Four pure moments (flexion: 8 N·m, extension 6 N·m, lateral bending ± 6 N·m, rotation ± 4 N·m) were applied to the centre of the upper surface of L1 to simulate the motion of lumbar spine. We defined the motion of the sagittal, coronal and transverse planes as flexion and extension, lateral bending and rotation, respectively. The range of motion (ROM) of each segment was compared with the results of previous studies. In addition to verifying the ROM of each segment of the lumbar model, we also verified the intervertebral disc pressure (IDP) of the L4/5 segment. Referring to previous studies by Brinckmann et al., the IDP of L4/5 segment was measured by applying a gradually increasing compression force (300N, 1000N) [43, 44].

### Boundary and loading conditions

We used ABAQUS software for the final model analysis calculations. We imported the model components into the software in INP format to assemble the model, and then constrained the boundary of the model and loaded the load at the same time. We imposed the same conditions on the intact model and surgical models, constrained the base of the sacrum, restricted its movement in all directions, and applied an axial load of 280 N on the L1 vertebral body to simulate part of the human weight [27, 39]. A moment of 7.5 N·m was applied to simulate the flexion, extension, lateral bending and rotation of the lumbar spine [22].

### Assessment indexes

In this study, the biomechanical characteristics between different models were compared by calculating and measuring the ROM (flexion, extension, tilt, rotation) of the fixed and cephalic adjacent segment, the stress of screw-rod system, the stress at the interface between cage and L5 endplate, and IDP of the adjacent segment. To analyze and compare the advantages and disadvantages of different surgical methods, so as to provide theoretical basis for clinical work.

## Results

### FE model validation

**Fig. 3:**
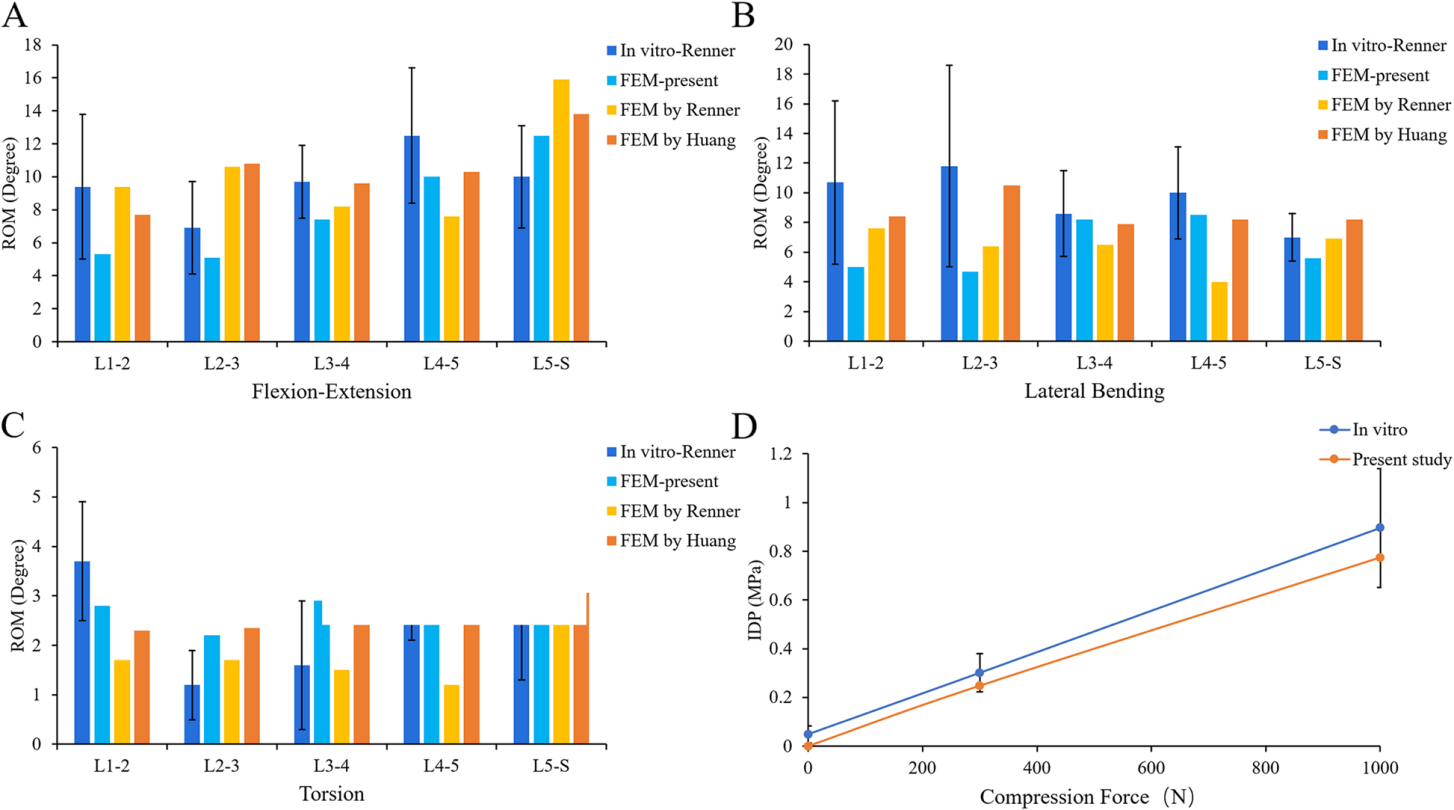
Comparison of the IDP of L4/5 and the ROM of each motion segment between the present and previous studies

Combined with previous experimental studies, this study verified the rationality of this lumbar spine model from two aspects [34, 42, 44]. On the one hand, the ROM of each lumbar motion segment was compared, and on the other hand, the IDP of the L4/5 segment was verified (Fig. 3). The measured ROM of each segment was in good agreement with previous in vitro experiments and FE studies. The L4/5 IDP also showed the same increasing trend as the previous in vitro experiments under gradually increasing compression load. The results showed that the FE model of this study was consistent with the physiological characteristics of the human body, and was effective for our next research.

### The ROM of the fixed segment

**Fig. 4:**
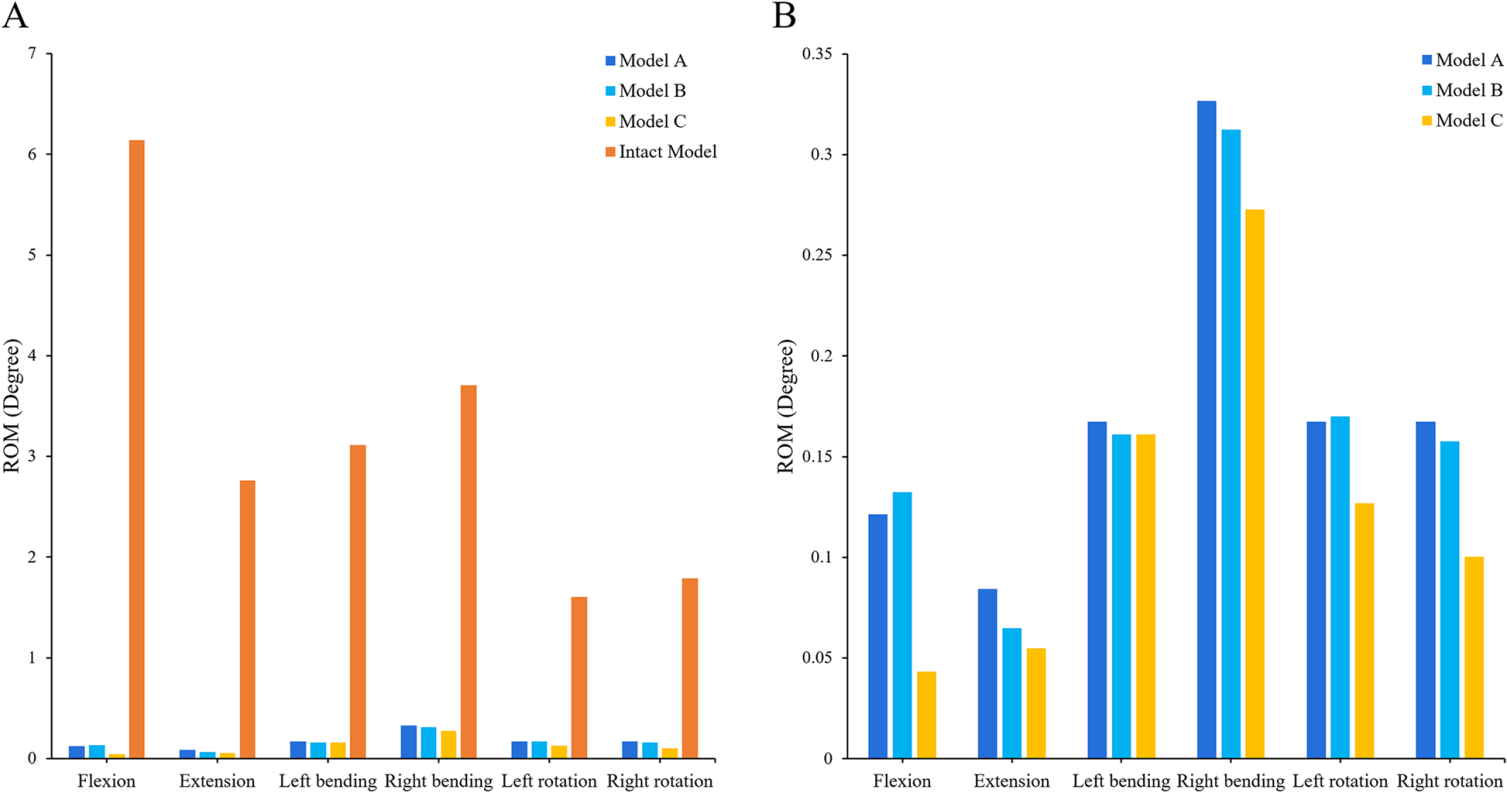
Comparison of the ROM at the fusion segment. **A** Between the intact and surgical FE models. **B** Between the different surgical FE models

The ROM of each surgical model and intact model in the fixed segment is shown in Fig. 4. The results showed that the internal fixation device provided obvious fixation effect, the surgical models showed a good limiting effect on the fusion segment and the ROM in six directions was significantly smaller than that in the intact model. Among them, the motion of the surgical models was most limited in the sagittal plane, and the least in the extension. The ROM of the fixed segment in the surgical models decreased by 99.3% in flexion. Although the ROM in the coronal plane of the surgical models was greater than that in the other directions, compared with the intact model, the motion limitation in the transverse plane (91.8%) was less than that in the coronal plane (94.0%). And the ROM of different surgical models had a good trend in the fixed segment. On the whole, the ROM of model A was the highest, while that of model C was the least. In the state of flexion, the ROM of Model C was only 35.7% of that of model A.

### ROM of the cephalic adjacent segment

**Fig. 5:**
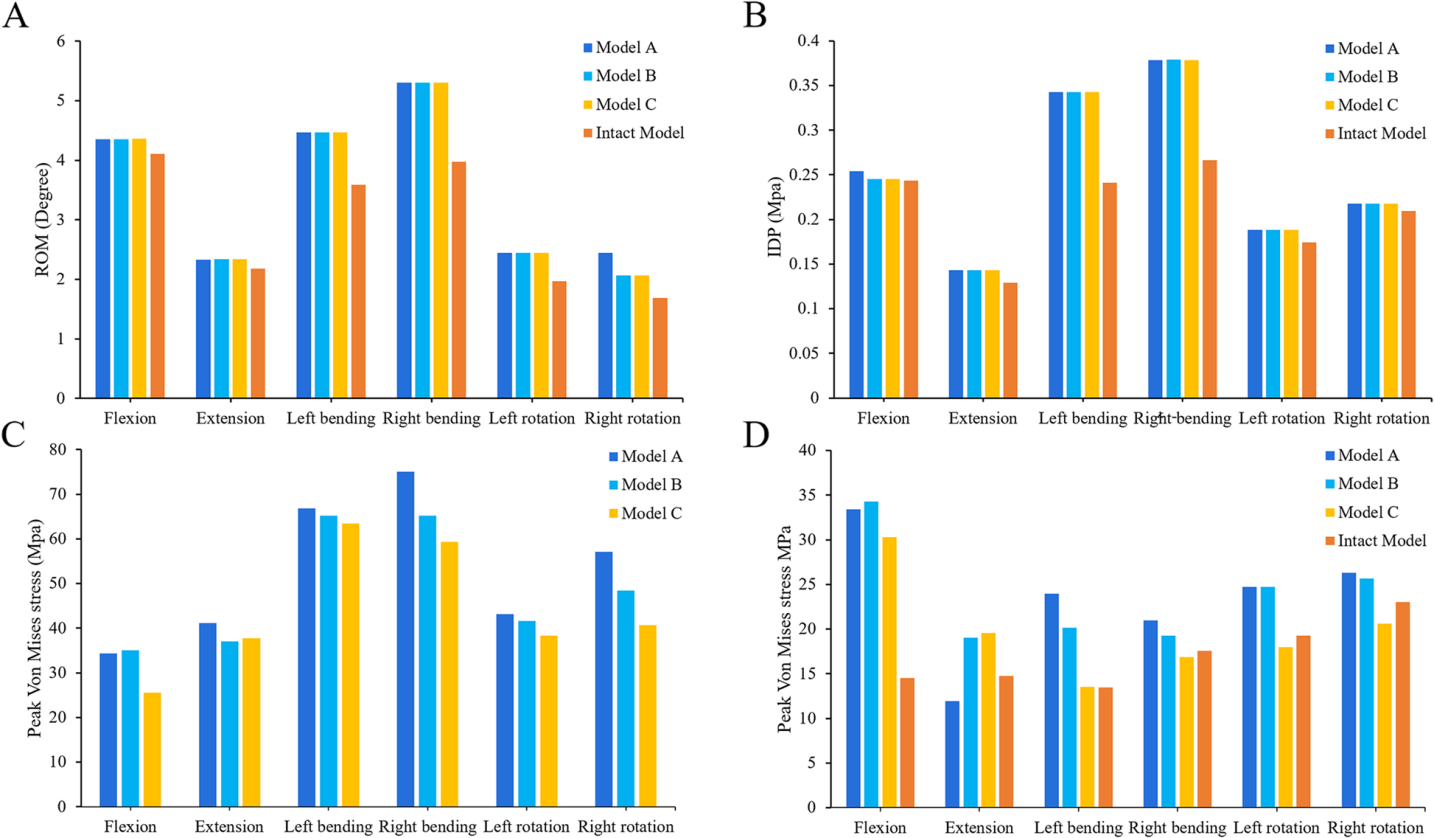
Comparison of calculation results of each structure in the different models. **A** The ROM of the cephalic adjacent segment **B** the IDP of the L3/4 segment **C** The stress of the screw-rod system **D** The stress of the cage-endplate interface

As shown in Fig. 5A, the ROM of the intact model was smaller than that of the surgical models in all directions. This phenomenon was most obvious in the lateral bending, in which the difference between the surgical models and the intact model was the largest (1.3°) in the right bengding, while the minimum difference in extension was only 0.2°. Although the ROM of the surgical models was the highest in the lateral bending and the smallest in the rotation, there was no significant difference in the ROM of each surgical model in all directions (less than 0.1°), except that the model A in the right rotation was 0.4° higher than that of other surgical models.

### IDP of the adjacent segment

The IDP of the adjacent segment of the intact model and surgical models is shown in Fig. 5B. The results showed that the IDP of all surgical models was greater than that of the intact model in the adjacent segment of L4/5, especially in the right bending, but the difference was the smallest in the right rotation. On the whole, the IDP of the L3/4 segment of the surgical model was the largest in the coronal plane, the second in the sagittal plane and the smallest in the transverse plane. Although the IDP of the adjacent segment of surgical models was slightly larger than that of the intact model, the difference of IDP between the surgical models was not significant.

### Stress of the screw-rod system

The stress distribution of the internal fixation system provides a basis for us to evaluate postoperative screw rod fracture, loosening and other complications. As shown in Fig. 5C, the stress of screw rod in lateral bending was the highest, while the stress in flexion–extension was the lowest, and the rotation was between them. On the whole, they showed a good trend in all directions. The stress of model A was the highest and that of model C was the lowest. Among them, the stress of model A was the largest in the right bending, up to 75.1 MPa, and the difference between model C and model A was 16.1 MPa, while the difference between them was the smallest in the extension, which is only 3.3 MPa. Although the stress of model B was higher than that of model A in flexion, the difference between them was only 0.7 MPa.

### Stress of the cage-endplate interface

**Fig. 6:**
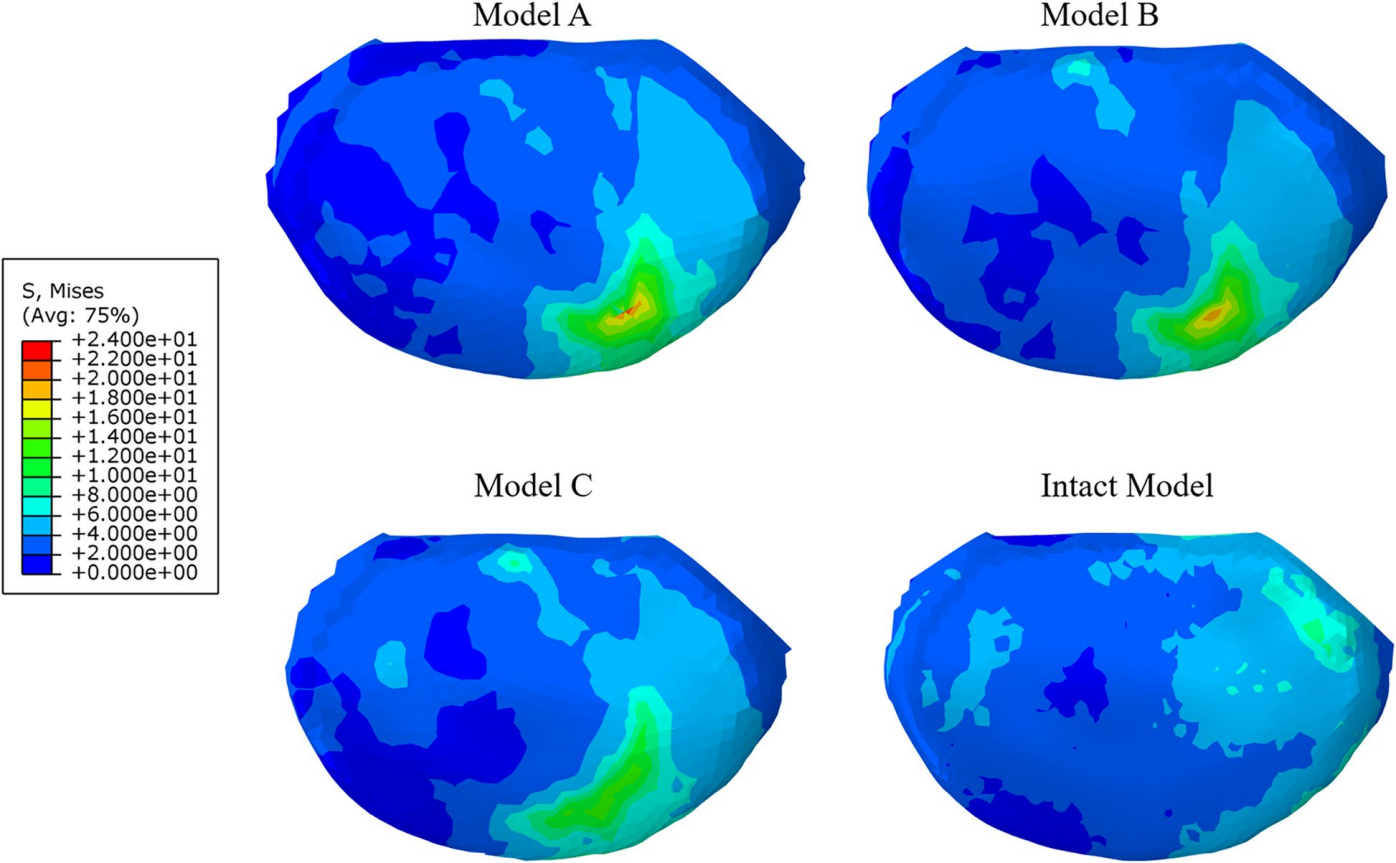
The stress distribution of the cage-endplate interface of different models during left bending

On the whole, the stress of L5 upper endplate of each surgical model was larger than that of the intact model (Figs. 5D and 6). Except in the sagittal plane, the endplate stress of model A was greater than that of model B. The maximum stress of the L5 upper endplate reached 34.3 MPa (model B) in flexion, which was only 0.9 MPa different from that of model A, but 2.4 times that of the intact model. In extension, the endplate stress of model A was only 11.9 MPa, which was not only smaller than that of surgical models, but also smaller than that of the intact model (2.8 MPa). Except for extension, the endplate stress of model C was less than that of other surgical models, and the minimum stress was only 13.5 MPa in left bending. Moreover, model C was the most similar to the intact model in the stress of the endplate compared with other surgical models.

## Discussion

Although great progress has been made in the surgical technique of interbody fusion with the development of medical technology, postoperative complications such as non-union, pseudarthrosis and cage subsidence are still a major challenge for surgeons [4]. Therefore, it requires surgeons to improve their surgical techniques, develop better surgical instruments, and more ideal biological agents. Although previous experiments have found that different sizes of cage may affect the possibility of cage subsidence [15], but not only its own design has limitations, and there is no further biomechanical study of each structure. In this study, FE analysis was used to evaluate the mechanical characteristics of various tissue structures of interbody fusion with different sizes of 3D printed cage.

Because of the characteristics of FE analysis, we could not judge whether the cage was fused to the vertebral body from the imaging data. According to FDA, the criteria for successful interbody fusion are as follow: the translation motion less than 3 mm and angular motion less than 5° [45]. In this study, the ROM of all surgical models in the fusion segment was far less than 5°, which was considered to achieve a good immediate postoperative fixation effect. Cage A (9 × 10 × 23 mm) is the most commonly used cage in PLIF. The axial area of the other two cages relative to cage A is 113% (cage B) and 167% (cage C), respectively. Although the ROM of each surgical model in the fusion segment was small, it also showed a trend of change. With the increase of the axial area of the cage, the ROM of the surgical models at the fixed segment showed a trend of decreasing. Although this was only a static mechanical result, it also reflected a changing trend. A larger contact area means that we can fill more bone grafts and better promote bone fusion in well-fixed segments. Because LLIF allows larger sizes of cage, the results of comparing LLIT and TLIF showed that LLIF provided better stability than TLIF, and its long-term postoperative cage subsidence was lower, which was consistent with our results [46, 47].

Rigid fixation of the motion segments may cause the loss of normal activity, resulting in the compensatory increase of the ROM and IDP of the adjacent segment, thus accelerating degeneration and increasing the risk of ASD [48, 49]. In this study, the ROM and IDP of the cephalic adjacent segment of surgical models were larger than those of the intact model. However, there was no significant difference in the ROM and IDP between surgical models. Previous studies on the pathogenic factors of ASD have found that decompression of non-fusion segments, the level of fusion segments and the degree of degeneration of adjacent segments highly affect the occurrence of ASD, while the surgical approach and the use of instruments including interbody fusion devices and pedicle screws do not increase the incidence of ASD [50, 51]. In addition, the 3D printed cage was used in this study to achieve an ideal geometric contact between the Cage and the vertebral body, and pedicle screws were used to achieve rigid fixation. Therefore, even though the interbody fusion in this study increased the ROM and IDP of adjacent segment after the application of different sizes of cage, there was no difference in the incidence of ASD between them.

As a long-term postoperative complication, the cage subsidence reduces the intervertebral space height of the fusion segment to a certain extent, weakens the support of the anterior column, and increases the load-bearing pressure of the posterior approach, which leads to recurrent low back pain and nervous system symptoms, failure of internal fixation and increase of reoperation rate [10, 52]. The stress of cage-endplate interface is the main factor leading to cage subsidence. The stress of surgical models at the interface between cage and endplate is greater than that of the intact model. In addition to the movement in the sagittal plane, the stress of the endplate between the surgical models had a certain trend, which was more obvious in lateral bending. However, the endplate stress of model C was most close to that of the intact model. This may be due to the fact that the larger cage has a larger contact area under the same load, which can disperse the pressure over a larger area, thereby reducing the pressure on the endplate, which is consistent with the previous research results [15]. The stress of the endplate of surgical models was much higher than that of the intact model in flexion, and the maximum stress reached 34.3 MPa, which was 2.4 times that of the intact model. Although the endplate stress of model C was smaller than that of other surgical models, it was still as high as 30.3 MPa, which was 2.1 times that of the intact model. Although the stress of endplate of surgical models is much less than the destructive strength of the cortical bone (90–200 MPa) [38], our analysis is based on a specific condition, while the real human activity is more complex. However, our results reflect such a trend that the endplate stress of cage with larger contact area is lower.

Previous studies have indicated that that the application of interbody fusion cage and screw-rod system establishes an effective stress conduction pathway, which makes the stress of internal fixation system dispersed [36]. The use of interbody fusion cage can bear more pressure in the anterior column and reduce the stress of the screw-rod system, which is also confirmed by our study results. In our study, the stress of the screw-rod system showed a decreasing trend in surgical models as the axial area of cage increased. The larger contact area has a higher load-bearing effect on the front column and can better disperse the pressure of the internal fixation system. Although the use of cage greatly reduced the stress of the screw-rod system, the maximum stress of the model screw-rod system of surgical models was only 75.1 MPa, which was far less than the yield strength of titanium (825–895 MPa) [38]. However, as mentioned above, under the complex movements in daily life, the stress of the internal fixation system may be further increased, resulting in the risk of internal fixation failure and screw rod fracture. However, the stress trend of the screw-rod system of surgical models also brings some enlightenment to us.

There are some limitations in our research. Firstly, the data of this study were based on a 24-year-old adult male and was not statistically analysed, which may have the possibility of individual differences, which is a common defect of finite element analysis. Secondly, we simplified FE model, and the material properties of each structure were assumed to be isotropic, which cannot more accurately reflect the biomechanical changes in lumbar structure. Therefore, we will pay more attention to the characteristics of materials in future research. Moreover, we did not simulate the complex changes brought about by muscles, which did not more accurately reflect the normal physiological movement of the lumbar spine. Finally, it is a pity that the data in this study were based on normal bone population and did not consider osteoporosis population. In the future, we plan to conduct more reasonable and rigorous biomechanical studies to verify our results.

## Conclusions

Cage with larger axial area in interbody fusion can reduce the stress of internal fixation system and the stress of the interface between cage and endplate, and has a certain effect in preventing cage subsidence, internal fixation system failure and screw rod fracture. Cage with larger axial area will not lead to an increase in the ROM and IDP of adjacent segment, and promote the occurrence of ASD.

## Data Availability

The datasets used and/or analysed during the current study available from the corresponding author on reasonable request.

## Abbreviations

FE: Finite element
ROM: Ranges of motion
IDP: Intradiscal pressure
ASD: Adjacent segment disease
PLIF: Posterior lumbar interbody fusion
LLIF: Lateral lumbar interbody fusion
TLIF: Transforminal lumbar interbody fusion
